# Inertial Sensor Reliability and Validity Across a Five-Level Surface Instability Gradation During Single-Leg Standing

**DOI:** 10.3390/s26113575

**Published:** 2026-06-04

**Authors:** Fani Paderi, Analina Emmanouil, Konstantinos Boudolos, Elissavet Rousanoglou

**Affiliations:** School of Physical Education and Sport Science, National and Kapodistrian University of Athens, 172 37 Daphne, Greece; fanipant@phed.uoa.gr (F.P.); cbountol@phed.uoa.gr (K.B.); erousan@phed.uoa.gr (E.R.)

**Keywords:** postural stability, inertial sensors, force plate, concurrent validity, balance graduation, shank kinematics, Bland–Altman

## Abstract

**Highlights:**

**What are the main findings?**

- Superior Reliability of Inertial Sensors: The inertial sensor unit demonstrated higher internal consistency and faster stabilization (excellent reliability ICC > 0.95 with lower trials) across increasing levels of instability compared to the force-plate.
- Identification of Mechanical Decoupling: Analysis of standardized metrics reveals that while inertial sensors and force plates are globally concordant, they capture complementary components of balance (correction effort vs. displacement), particularly during conditions of intensified instability (i.e., BOSU). Both devices agree on the postural challenge graduation (Global Concordance) as well as how stable a specific person is relative to the group (Individual Ranking Agreement).

**What are the implications of the main findings?**

- Validation of a Five-Level Postural Challenge Graduation: The study establishes a reliable and valid surface graduation for single-leg balance, providing a research-based framework for progressive rehabilitation and athletic training.
- Global Concordance—Individual Ranking Agreement: Both inertial sensors and the force plate metrics agree on the postural challenge graduation (Global Concordance) as well as on how stable a specific person is relative to the group (Individual Ranking Agreement).

**Abstract:**

Wearable inertial sensors offer a portable alternative to laboratory-grade force plates for postural stability assessment; however, their validity across progressively challenging balance tasks remains under-explored. This study evaluated the reliability and concurrent validity of inertially sensed metrics compared with force-plate-derived postural sway metrics across a five-level spectrum of unstable surfaces (Floor, Foam Pad, Rotating Disc, Air Disc, Bosu). Twenty-five healthy young women (22.1 ± 3.6 years, 1.64 ± 0.04 m, 58.44 ± 8.21 kg) performed five trials of single-leg standing (40 s each) on each surface. Postural sway was computed from antero-posterior (AP) and medio-lateral (ML) center of pressure (CoP) recordings using a force plate (Kistler, 9286 AA, Winterthur, Switzerland, sampling at 500 Hz) in synchronization with a lateral shank-mounted inertial sensor (Bionomadix BN-ACCL3, Biopac Systems, Inc., Santa Barbara, CA, USA, sampling at 100 Hz). In addition to reliability, a two-tiered analysis evaluated global concordance (unstandardized slopes) and method agreement (standardized z-scores). Intraclass correlation coefficients (ICCs) for the inertial sensor were excellent (range: 0.95–0.96), surpassing the force plate (range: 0.85–0.92) as trials accumulated. Analysis revealed moderate-to-good global concordance in the AP direction (*r* = 0.60, *p* = 0.001) and good-to-excellent in the ML one (*r* = 0.85, *p* < 0.001), validating the progressive intensifying effect of the surface graduation. Individual ranking agreement—evaluated via standardized z-scores—was also significant in both the AP (*r* = 0.61, *p* < 0.001) and the ML (r = 0.85, *p* < 0.001) directions, indicating a convergence into how the two modalities rank individual performance. Bland–Altman plots confirmed high absolute agreement between standardized scores, though a predictable proportional bias was observed in raw units, where the inertial sensor’s underestimation of sway magnitude increased linearly with task difficulty. The five-level postural challenge graduation is a highly reliable framework for balance assessment. While the shank-mounted sensor exhibits proportional underestimation of sway magnitude compared to the CoP at extreme intensities, its high internal stability and sensitivity to task difficulty make it a valid and robust tool for longitudinal clinical monitoring.

## 1. Introduction

Postural stability assessment is a cornerstone of clinical rehabilitation and athletic performance monitoring. Currently, Center of Pressure (CoP) recording via a force plate remains the established “gold standard” for quantifying postural sway [1,2]. However, the high cost and lack of portability of force plates have led to the increasing use of wearable inertial measurement units. These sensors, typically integrating accelerometers and gyroscopes, offer a versatile alternative for field-based stability studies, particularly those involving environmental challenges like unstable surfaces [3,4].

Research has validated inertial sensors against CoP measures across various standing tasks. In bipedal standing, inertial sensor-derived metrics demonstrate good-to-excellent concurrent validity (r > 0.75) [5]. While correlations remain strong for single-leg standing (r ≈ 0.79) [4], they tend to moderate during more complex tasks like tandem stance (r = 0.430–0.493) [6]. However, it is well established that force plates and inertial sensors capture distinct physical components of balance—CoP displacement (m) versus shank acceleration (m/s^2^) [7,8]. While force plates are highly sensitive to slow, global postural shifts, inertial sensors capture fast, jerky corrective joint accelerations [9]. Furthermore, sensor placement on lower limb segments, such as the lateral shank, has been proposed as a method for isolating ankle-specific postural strategies [9].

A common method for challenging postural control involves single-leg standing on unstable surfaces [10,11,12], which amplifies the demand on ankle dynamics and the surrounding musculature [13]. In clinical and athletic training, a diverse array of tools is utilized to induce this instability, including deformable surfaces (e.g., foam pads), which dampen proprioceptive feedback [14], air-filled surfaces (e.g., air discs), providing multi-directional instability [15], and rigid hemispheric platforms (e.g., BOSU, acronym for Both Sides Utilized), which create large displacement arcs demanding marked corrective tibio-tarsal joint motions [16,17].

Practitioners often prescribe balance progressions based on anecdotal experience rather than research-based evidence regarding the postural challenge gradation of commonly used unstable surfaces. Literature appears limited to pairing a stable ground reference against just a single unstable surface (most commonly a foam pad) [10,11,12], an issue already noted by Neville et al. [4]. To the best of our knowledge, there are no studies evaluating a comprehensive, multi-level spectrum of unstable surfaces within a single experimental protocol. This lack of comparative data deprives practitioners of the objective, research-based evidence required to design structured progressive rehabilitation programs. Quantifying the specific postural challenge of each surface would provide significant practical value, offering research-based evidence for clinicians aiming to systematically increase postural demand, while verifying that inertial sensors can reliably differentiate between these varying levels of instability is essential for field-based monitoring.

Therefore, the purpose of this study was to evaluate the reliability and concurrent validity of inertially sensed postural stability metrics against force plate-derived metrics during single-leg standing across a five-level spectrum of commonly used unstable surfaces. Based on the distinct mechanical profiles of the unstable surfaces [16,17] and the measurement sensitivity of both modalities [7], the following hypotheses (H) were tested:

**H1 (Method Reliability):**  *Both the force plate CoP metrics and the inertial sensor acceleration (Acc) metrics will demonstrate high reliability (ICC > 0.75) in the anteroposterior (AP), as well as in the mediolateral (ML) directions across all conditions*.

**H2 (Postural Challenge Gradation):**  *Postural sway magnitude, measured by both force plate CoP metrics and Acc metrics, will increase significantly and linearly across the five levels of the postural challenge gradation*.

**H3 (Global Concordance vs. Individual Ranking Agreement):**  *There will be a strong positive correlation (r > 0.70) between the unstandardized Acc and CoP slope coefficients across the surface progression (Global Concordance) as well as between the standardized individual scores within each surface (Individual Ranking Agreement) although the two devices measure different physical entities (displacement vs. acceleration) [18]*.

**H4 (Method Agreement):**  *Bland–Altman analysis will reveal a proportional bias between the standardized scores of the two devices, with the inertial sensor progressively underestimating sway magnitude as the task difficulty increases*.

## 2. Materials and Methods

### 2.1. Participants

Twenty-five young women (age: 22.1 ± 3.6 years, body height: 1.64 ± 0.04 m, body mass: 58.44 ± 8.21 kg and BMI 21.6 ± 2.5 kg/m^2^) participated in the study. Only women with normal BMI (from 18.5 kg/m^2^ to 24.9 kg/m^2^) and within a height range from 1.60 m to 1.75 m were recruited for the study. By utilizing a single-sex cohort, we aimed to maximize internal validity and isolate sensor-derived stability indices from the ‘noise’ of -sex-related physiological “noise”—such as variations in center-of-mass height, limb morphology, and neuromuscular control strategies [19]—thereby maximizing the internal validity of the sensor-based validation metrics. Exclusion criteria were the existence of musculoskeletal injuries, neurological disorders or any dysfunction of the vestibular and visual system, as registered in a self-reported screening questionnaire. The study was approved by the Bioethics committee of the School of Physical Education and Sport Science, National and Kapodistrian University of Athens, Greece (Approval Protocol Number: 1497/15-03-2023). Each participant signed an individual consent form.

### 2.2. Experimental Procedure

**Figure 1:**
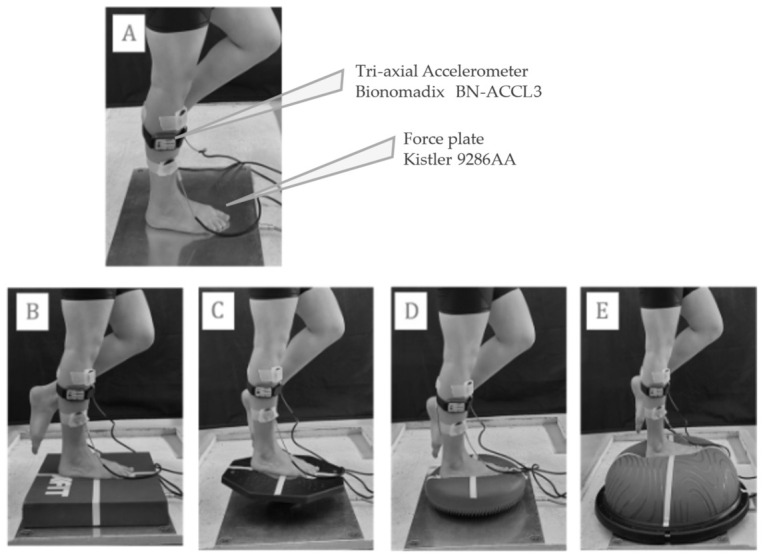
Single leg-standing on the five different surfaces of (**A**) Floor (standing on the force-plate), (**B**) Foam Pad, (**C**) Rotating Disc, (**D**) Air Disc, and (**E**) BOSU. Experimental set-up. All unstable surfaces were positioned on the force-plate by aligning the geometrical center of the device with the center of the force plate. The accelerometer used to collect the linear acceleration data was securely positioned at the lateral side of the lower leg, at a position approximating the segmental center of mass. Τhe anatomical calibration of the sensor is illustrated (top). The AP movement is indicated with the Ax-axis concerning the CoP path data and the X-axis concerning the accelerometry data. The ML movement is indicated with the Ay-axis concerning the CoP path data and the Z-axis concerning the accelerometry data. The Y-axis of the accelerometer indicates vertical movement; however, only the AP and ML directions were examined in the present study.

Participants performed five trials of single-legged stance while standing on five different surfaces (Figure 1) (Floor, Foam Pad, Rotating Disc, Air Disc, BOSU). The surface height was measured both at the beginning and at the end of the measurements to check that the air pressure level was not altered [20]. The participants stood barefoot with their dominant foot positioned at the geometrical center of the standing surface, so that every trial could be started at the same foot configuration. The participants were asked to place their hands on their hips and ensure that their heads were facing forward. Furthermore, they were instructed to keep their gaze fixed on a target (a red circle) that was positioned in front of them at eye level and at 2 m horizontal distance, to minimize any differential visual effect [5]. The correct repositioning of each participant’s dominant foot was carefully checked. The recording duration was 40 s with a 2-min break between trials, and a 5-min rest period between surfaces. The order of the surfaces was counterbalanced using a systematic rotational method. While the specific sequence of surfaces remained constant, each subsequent participant started on the surface immediately following the one used by the previous participant. Thus, a balanced design was achieved where an equal number of participants (N = 5) initiated the protocol on each respective surface.

### 2.3. Data Collection and Analysis

#### 2.3.1. Inertial Sensor Data

A triaxial accelerometer (BioNomadix BN-ACCL3, Biopac Systems, Inc., Santa Barbara, CA, USA), sampling at 100 Hz (AcqKnowledge 5.0 software, Biopac Systems, Inc., Santa Barbara, CA, USA), was used to collect the 3D linear acceleration time series. The sensor was securely attached to the lateral aspect of the shank at about the segmental center of mass (42% of shank length as measured from the lateral femoral condyle to the lateral malleolus [21]). Acceleration signals were recorded in three directions: anteroposterior (AP), mediolateral (ML), and vertical (Vert). An anatomical calibration procedure ensured that the X, Z, and Y axes corresponded to AP, ML and Vert directions of shank motion, respectively (Figure 1A); however, only the AP and ML directions were examined in the present study. For each trial and each surface condition (Floor, Foam Pad, Rotating Disc, Air Disc, and BOSU), the acceleration signals were filtered using a fourth-order Butterworth low-pass filter with a 10 Hz cut-off frequency. From the standardized 40-s recordings, the first 5 s were excluded to minimize start-up artifacts, and the remaining 30 s of valid data per trial were used for the path calculations. Subsequently, acceleration path length (Acc-path) was calculated for AP and ML directions (Acc-AP and Acc-ML, respectively), as the cumulative sum of the absolute differences between successive acceleration data points, representing the total excursion of the shank acceleration signal during the single-leg stance task.

#### 2.3.2. Force Plate Data

In synchronization with the inertial sensor, a force plate (60 × 40 × 3.5 cm, type 9286AA, Kistler, Switzerland), sampling at 500 Hz (BioWare^®^ v3.2.6.104, Kistler, Switzerland), was used to collect kinetic data of the center of pressure (CoP) trajectory in the AP and ML directions. The force plate was synchronized with the accelerometer via an analog interface cable (CBL102), linking the Kistler data acquisition system (DAQ-Kistler 5691 A1, Kistler, Switzerland) to the BIOPAC MP150 main unit (Biopac Systems, Inc., Santa Barbara, CA, USA). A trigger signal generated by the accelerometer initiated simultaneous data acquisition in both systems, ensuring precise temporal alignment. For each trial and each surface condition, the CoP signals were filtered using a fourth-order IIR Butterworth low-pass filter with a 10 Hz cut-off frequency (MATLAB R2025a, MathWorks, Natick, MA, USA). As with the accelerometer data, the first 5 s of each 40-s recording was excluded, and the remaining 30 s of valid data were retained for analysis. CoP path length (CoP-path) was then calculated separately in the AP (CoP-AP) and ML (CoP-ML) directions, as the cumulative sum of successive CoP displacements during each trial.

#### 2.3.3. Reliability Analysis

A reliability analysis (Table A1) was applied to decide the optimum number of trials’ accumulation for testing the study hypothesis using the ICC, SMD%, SEM%, and CV% measures [22,23,24,25,26,27,28]. Overall, the results of accumulated reliability analysis (Table A1) indicate that inertial sensor–derived measures exhibit higher reliability and lower measurement error compared to force plate measures, particularly in the AP direction. Across both measurement systems, the accumulation of trials led to improved reliability, with four to five trials providing the most stable estimates. Based on these findings, the five-trials average was decided for the computation of the slope coefficient used in subsequent statistical analyses.

#### 2.3.4. Slope Coefficient

The individual slope coefficient (β) derived from linear regression analysis was defined as the primary outcome variable of the study, used to quantify the performance modulation across the graduation of surface instability. For each participant, a linear curve was fitted across the five surface conditions using MATLAB R2025a (MathWorks, Natick, MA, USA). In these individual regression models, the independent predictor variable consisted of the five standing surfaces assigned to consecutive ordinal levels, representing the conceptual progression of difficulty (postural challenge graduation) as described in Section 2.4.2.

The dependent variables were the five-trials average of each postural sway metric (Acc-AP, Acc-ML, CoP-AP, CoP-ML). The resulting unstandardized slope coefficient (β) quantifies the exact rate of change in shank acceleration or CoP displacement as surface instability intensifies. A higher β value indicates greater sensitivity and a more pronounced postural reaction to the incremental balance demands. The derived individual slope coefficients were the metrics used for the concurrent validity and the method agreement analyses described in Section 2.4.3.

### 2.4. Statistical Analysis

All descriptive values are presented as means standard deviations. Statistical analyses were performed using SPSS v30.0 (IBM Corp., Armonk, NY, USA), with the alpha significance threshold set at 0.05, against which all produced *p*-values were evaluated.

#### 2.4.1. Preliminary Screening

Data were screened for outliers and normality. Standardized z-scores identified only one observation slightly exceeding the traditional outlier threshold of 3.29 (for Foam Pad in Acc-ML); this participant was retained to preserve sample representativeness. While Shapiro–Wilk tests indicated non-normality in three ML acceleration parameters (*p* < 0.05), the skewness (<|1.7|) and kurtosis |6.0| for all variables remained within acceptable behavioral limits.

#### 2.4.2. Validation of Postural Challenge Graduation

To confirm that the five surfaces represented a progressively intensifying balance challenge, individual slope coefficients (unstandardized slopes) were calculated across the ordered surfaces (Floor, Foam Pad, Rotating Disc, Air Disc, BOSU). A one-way repeated measures ANOVA evaluated the main effect of surface instability. The Greenhouse–Geisser correction was applied in cases where the assumption of sphericity was violated. To track sequential transitions between specific surface tiers, planned repeated within-subjects contrasts were utilized to evaluate the overall linear trend of the postural graduation [29,30]. By focusing on these specific transitions, this method provides greater statistical power and mitigates Type I error inflation [31]. Effect sizes were calculated and interpreted according to standard benchmarks to quantify the magnitude of the postural modulation [31,32].

#### 2.4.3. Concurrent Validity Analysis

The concurrent validity between the inertial sensor and the force plate slope coefficients was evaluated using a two-tiered correlation approach (global trend concordance and individual ranking agreement). In both approaches, the Pearson’s values were interpreted according to Portney and Watkins [22] as: little or no correlation (*r* = 0.00–0.25), poor (*r* = 0.25–0.50), moderate-to-good (*r* = 0.50–0.75), good-to-excellent (*r* > 0.75).

- Global Trend Concordance: Bivariate Pearson’s correlations were calculated using unstandardized slope values. This assessed the mutual sensitivity of both devices to track the systematic, global increase in sway magnitude across the surface progression.
- Individual Ranking Agreement: To evaluate device agreement at the individual subject level independent of task intensity, slopes were transformed into standardized z-scores based on the group mean and standard deviation. This z-score transformation was methodologically necessary to eliminate the profound scaling differences between displacement (m) and acceleration (m/s^2^) units. Without standardization, the shared variance of the progressive surface difficulty would artificially inflate the correlation. Standardizing the metrics isolated the devices’ capacity to rank individual performance consistently.

#### 2.4.4. Method Agreement Analysis

Fixed systematic bias between the standardized slope coefficients of the two modalities was evaluated using paired-samples *t*-tests for both the AP and ML directions. To establish absolute agreement, Bland–Altman plots were constructed using these standardized scores. The mean difference (fixed bias) and the 95% limits of agreement (LoA; mean difference ± 1.96 times SD of the differences) were calculated to quantify the absolute limits of agreement and identify the presence of proportional bias across the spectrum of task difficulty.

## 3. Results

### 3.1. Validation of the Postural Challenge Gradation

The repeated measures ANOVA confirmed a significant main effect of surface type across all evaluated stability metrics: Acc-AP (F(4, 96) = 113.4, *p* < 0.001, partial *η*^2^ = 0.825), Acc-ML (F(4, 96) = 114.18, *p* < 0.001, partial *η*^2^ = 0.826), CoP-AP (F(4, 96) = 64.47, *p* < 0.001, partial *η*^2^ = 0.729), and CoP-ML (F(4, 96) = 56.61, *p* < 0.001, partial *η*^2^ = 0.702).

**Figure 2:**
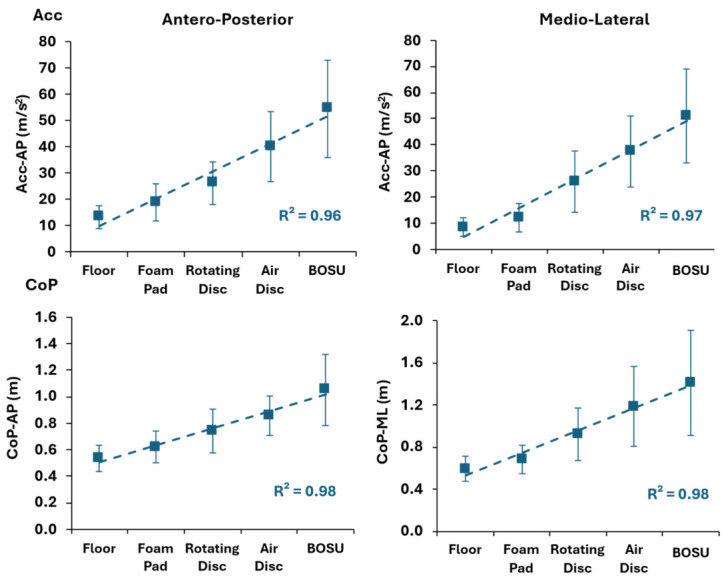
Estimated marginal means of postural stability across the five levels of the surface graduation. Dashed lines indicate the linear trendlines for each data series. Error bars represent the standard error of the mean (SE), and R^2^ indicates the explained variance for the linear component.

Trend analysis (Table A3) and planned repeated within-subjects contrasts (Table A4) revealed a predominant linear component (*p* < 0.001 for all metrics), validating the graduated scaling of the surfaces (Figure 2). In addition, a significant quadratic component (*p* < 0.05) (Table A4) highlighted the progressive intensification of task demands (*p* < 0.001 for all) (Figure 2), particularly during the transition from the Air Disc (Level 4) to the BOSU surface (Level 5) (Table A4).

### 3.2. Global Concordance

Global concordance analysis evaluated the macroscopic relationship between the two modalities using unstandardized slope coefficients across the full surface challenge. As expected due to dimensional scaling, unstandardized slope values were significantly lower in magnitude for the force plate than for the inertial sensor (*p* < 0.001).

**Figure 3:**
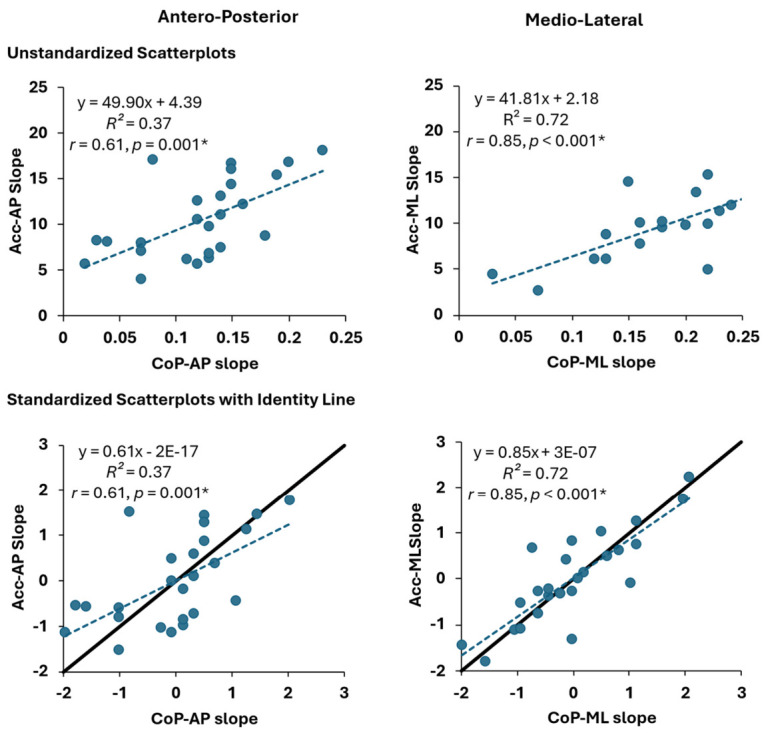
Concurrent validity scatterplots for unstandardized (**Top**) and standardized (**Bottom**) surface slopes between the inertial sensor (Acc) and force-plate (CoP) metrics, for the anterior–posterior (AP; **Left**) and medio-lateral (ML; **Right**) directions. *R*^2^ indicates the explained variance, *r* represents the Pearson correlation coefficient, and the solid black line denotes the identity line (y = x). The dashed lines represent the linear regression lines for each dataset. * Significant correlation at *p* < 0.05.

Despite this difference in scale, bivariate Pearson correlations revealed strong, positive global concordance between the two modalities (Figure 3–Top). The global trend relationship was moderate-to-good in the AP direction (*r* = 0.60, *p* = 0.001, Figure 3–Top Left) and good-to-excellent in the ML one (*r* = 0.85, *p* < 0.001, Figure 3—Top Right).

**Figure 4:**
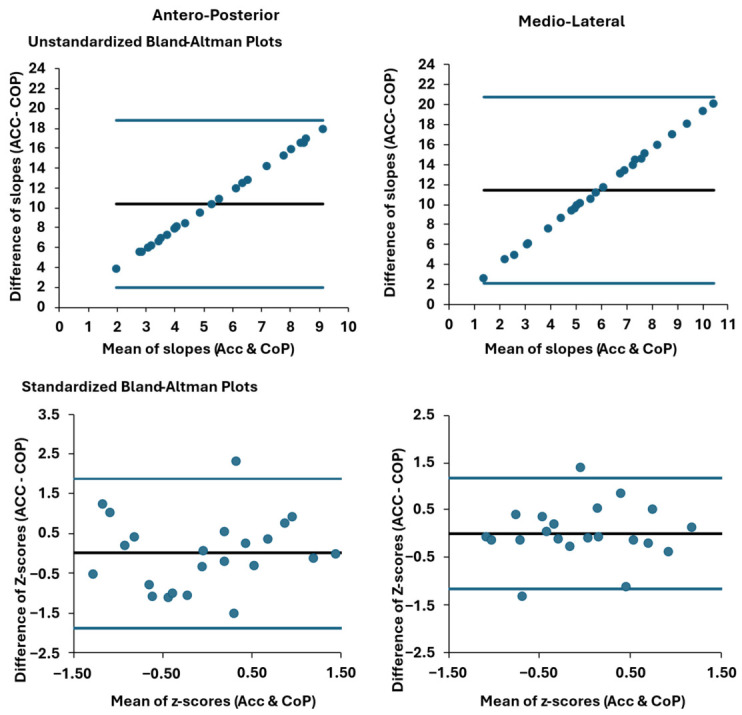
Bland–Altman plots validating the inertial sensor (Acc) against force-plate (CoP) metrics across a five-level postural challenge graduation in the AP (**Left**) and ML (**Right**) directions. (**Top**) Unstandardized plots evaluating slope values in raw units. (**Bottom**) Standardized plots evaluating method agreement independent of task magnitude (z-scores). The horizontal black lines indicate the mean bias, and the blue lines represent the 95% limits of agreement (LoA).

Bland–Altman plots for these unstandardized data (Figure 4, Top) illustrated a distinct and linear proportional bias. As the mean magnitude of postural sway increased along the X-axis, the absolute difference between the Acc and CoP metrics increased at a synchronized rate along the Y-axis. The lack of random scatter around this diagonal trend reflects the strong, parallel sensitivity of both devices to the expanding task difficulty, despite operating on different physical units (m vs. m/s^2^).

### 3.3. Individual Ranking Agreement

To assess the measurement agreement at the individual subject level independent of task intensity, slope coefficients were transformed into standardized z-scores. When evaluating individual ranking agreement, the correlation between the two modalities also reached statistical significance in both the AP and the ML directions.

In both the AP and the ML directions, individual data were distributed on both sides of the identity line (Figure 3 (Bottom)), indicating a lack of systematic bias. The correlation between the two modalities was moderate and statistically significant (*r* = 0.61, *p* = 0.001), with the linear regression explaining a minor portion of individual variance (*R*^2^ = 0.37). In the ML direction, the linear regression fit appeared visually tighter (*R*^2^ = 0.72), and the underlying correlation also achieved statistical significance (*r* = 0.85, *p* < 0.001).

The standardized Bland–Altman plots (Figure 4, Bottom) reflected these individual variations. After standardization, the mean bias was reduced to zero (0.00), and data points for the ML direction were tightly clustered within the 95% limits of agreement (LoA) (Figure 4, Bottom Right). The AP direction (Figure 4, Bottom Left) exhibited wider LoA and more pronounced outliers at higher sway magnitudes, indicating greater divergence between shank acceleration and center-of-pressure displacement during sagittal plane corrections.

## 4. Discussion

The present study demonstrates that the five-level surface spectrum successfully serves as a graduated postural challenge framework, characterized by distinct reliability profiles and measurement sensitivities between modalities. Rather than functioning as a direct 1:1 substitute for laboratory-grade force plates, the lateral shank-mounted inertial sensor captures a distinct, complementary dimension of postural control.

### 4.1. Reliability and Clinical Utility

A primary finding of this study was the exceptional reliability of the five-level graduation, particularly when utilizing the wearable inertial sensor. Based on the ICCs, the Acc metrics demonstrated excellent internal reliability (ICC ranging from 0.95 to 0.96) when aggregating data across the entire surface graduation. This profile proved highly repeatable when compared with the force plate metrics (ICC ranging from 0.85 to 0.92), suggesting that segment-acceleration data offers a stable representation of collective postural behavior across varying environmental challenges.

The trial-accumulation data provides clear boundaries for clinical translation. Reliability for both modalities improved systematically as the number of consecutive trials increased from one to five. Notably, the inertial sensor achieved acceptable stability (ICC > 0.80) within just two trials, whereas the force plate required additional exposures to reach comparable consistency. This high internal stability, combined with the established MDC (≈41–45%) and a declining SEM across cumulative trials, indicates that the sensor framework is a highly repeatable instrument well-suited for tracking macro-level progression during longitudinal balance rehabilitation.

### 4.2. Validity of the Postural Challenge Graduation

The structural validity of the five-level challenge is supported by the strong linear expansion of unstandardized sway values across the surface tiers in both the AP and the ML directions. Thus, despite the mechanical differences between unstable surfaces [16,17], this high global concordance confirms that both the force plate and the shank-mounted sensor possess the mutual sensitivity required to track the systematic escalation of task difficulty from the stable Floor to the highly unstable BOSU condition.

When removing the shared variance of task magnitude via standardized z-scores, individual ranking agreement was also at a significant level, indicating the measurement sensitivity convergence of the two devices also at subject-level, despite capturing distinct physical properties of postural control rather than interchangeable scores. As noted by Quijoux et al. [8], the force plate, tracking CoP displacement, captures the slow, low-frequency spatial outcome of a participant’s balance. Conversely, the shank-mounted sensor records high-frequency linear acceleration, which reflects the rapid, localized corrective actions taken by the segment to maintain equilibrium.

It must be noted that, as shown in Table A4, the surface graduation progresses with rising transitions that are not numerically equal. Yet, the difference of the rising transitions was not statistically significant, which validates the use of surface spectrum as the independent variable in the linear regression applied to compute the slope coefficient.

### 4.3. Directional Divergence and Hypothesized Mechanical Decoupling

A distinct finding within the standardized analysis was the superior agreement observed in the ML direction (*R*^2^ = 0.72) compared to the AP one (*R*^2^ = 0.37). This directional variance is highly consistent with the biomechanical constraints associated with a lateral shank sensor placement during single-leg standing. In the ML direction, single-leg balance involves a highly constrained lateral hip-and-pelvis strategy to keep the center of mass positioned over a narrow base of support. Because the shank acts as a relatively rigid lever in the frontal plane during these lateral shifts, the linear acceleration of the segment maps onto the force plate’s displacement metrics with high fidelity.

### 4.4. Limitations and Future Directions

While this study validates the five-level graduation, several limitations must be noted. First, the current study utilized a healthy, physically active cohort. The proportional bias and MDC values established here may differ in clinical populations (e.g., elderly fallers or individuals with vestibular disorders) who may exhibit different sway behaviors. However, focusing on a single-sex cohort controls for biological and biomechanical variability [19], thereby maximizing the internal validity of the sensor-based validation metrics. Secondly, we utilized a single-sensor setup (lateral side of the lower leg) to maximize clinical utility and portability, yet this specific placement is inherently more susceptible to local segment noise and multi-joint decoupling than center-of-mass or multi-sensor configurations.

Future research should compare lateral shank tracking directly with lumbar or multi-sensor arrays to clarify which configuration optimizes individual ranking agreement against a force plate. Additionally, exploring whether inertially sensed angular velocity can better characterize the high-frequency angular oscillations of the ankle may help improve directional concordance within the AP plane.

### 4.5. Conclusions

The five-level postural challenge graduation establishes a research-based reliable, valid, and objective framework for progressive rehabilitation and athletic training. The lateral shank-mounted sensor exhibits a predictable proportional bias in raw units—progressively underestimating sway magnitude at extreme task intensities due to suspected multi-joint mechanical decoupling. However, its high internal stability (superior reliability) and sensitivity to global task difficulty make it a highly practical, robust tool for longitudinal monitoring of global balance progress within a single individual over time. The two devices capture complementary components of balance (correction effort in inertial sensor vs. displacement in force plates), particularly during conditions of intensified instability (i.e., BOSU). Crucially, both inertial sensors and the force plate metrics agree on the postural challenge graduation (Global Concordance) as well as on how stable a specific person is relative to the group (Individual Ranking Agreement).

## Data Availability

Data is unavailable due ethical restrictions.

## Appendix A

### Reliability Analysis—Trial Accumulation

A reliability analysis (Table A1) was performed on the slope coefficients to determine the minimum number of trials required for stable slope estimates. The following reliability indices were computed:

(a) Intraclass Correlation Coefficient (ICC) using a two-way random-effects model for absolute agreement and average measures [21,22].
(b) Standard Error of Measurement (SEM) derived from the square root of the ANOVA residual mean square error (SEM = √S^2^_error) and its relative form expressed as a percentage of the mean (SEM% = (SEM/x) × 100) [23,24,25].
(c) Minimal Detectable Change at 95% confidence (MDC95), calculated from the SEM as MDC95 = SEM × 1.96 × √2, and its relative form expressed as a percentage of the mean (MDC95% = (MDC95/x) × 100) [23,24,25].
(d) Coefficient of Variation (CV%) calculated as the standard deviation of the trial-level slopes divided by their mean, multiplied by 100 (CV% = (SD/mean) × 100) [26,27].

The accumulated reliability analysis demonstrated a progressive improvement in reliability indices as the number of trials increased across all measures (Table A1).

For the slope coefficients derived from the inertial sensor (both Acc-AP and Acc-ML), reliability ranged from good to excellent even with two trials (ICC = 0.82–0.85) and improved to excellent levels with three or more trials (ICC ≥ 0.92). The highest reliability was observed with four and five trials (ICC = 0.94–0.96), accompanied by reduced measurement error, as reflected by lower SEM% (16.53–22.11%) and MDC% (38.58–51.58%). CV% also decreased with additional trials, indicating improved consistency.

For the slope coefficients derived from the force plate, CoP-ML showed good reliability with two trials (ICC = 0.79), improving to excellent reliability with three to five trials (ICC = 0.87–0.92), along with moderate reductions in SEM% (25.95–30.33%) and MDC% (60.56–70.77%). In contrast, CoP-AP demonstrated poor reliability with two trials (ICC = 0.38) and only reached acceptable to good levels with four to five trials (ICC = 0.76–0.85), while maintaining relatively high measurement error (SEM% = 38.70–45.92%; MDC% = 90.30–107.16%).

Overall, the results indicate that inertial sensor–derived measures exhibit higher reliability and lower measurement error compared to force plate measures, particularly in the anteroposterior direction. Across both measurement systems, the accumulation of trials led to improved reliability, with four to five trials providing the most stable estimates. Based on these findings, the five-trials average was selected for the computation of the final slope coefficient used in subsequent analyses.

**Table A1:** Reliability Analysis Indices.

Measure	Trial Accumulation	Number of Trials	ICC (95% CI)	SEM%	MDC95%	CV%
Acc-AP	1 to 2	2	0.84 (0.62, 0.93)	22.60	52.73	18.91
	1 to 3	3	0.93 (0.86, 0.97)	18.21	42.49	17.12
	1 to 4	4	0.96 (0.92, 0.98)	16.53	38.58	15.61
	1 to 5	5	0.96 (0.93, 0.98)	17.61	41.10	17.16
Acc-ML	1 to 2	2	0.85 (0.65, 0.93)	23.16	54.04	20.31
	1 to 3	3	0.93 (0.86, 0.97)	19.56	45.65	17.99
	1 to 4	4	0.95 (0.91, 0.98)	17.64	41.16	16.33
	1 to 5	5	0.95 (0.92, 0.98)	19.59	45.71	17.70
CoP-AP	1 to 2	2	0.38 (−0.48, 0.74)	45.92	107.16	40.07
	1 to 3	3	0.70 (0.41, 0.86)	39.41	91.96	36.37
	1 to 4	4	0.76 (0.56, 0.89)	41.30	96.36	37.16
	1 to 5	5	0.85 (0.72, 0.92)	38.70	90.30	34.26
CoP-ML	1 to 2	2	0.79 (0.50, 0.91)	30.33	70.77	24.29
	1 to 3	3	0.87 (0.74, 0.94)	26.72	62.35	25.41
	1 to 4	4	0.90 (0.82, 0.95)	26.04	60.77	22.97
	1 to 5	5	0.92 (0.86, 0.96)	25.95	60.56	24.22

ICC: Intraclass Correlation Coefficient, SEM%: Standard Error of Measurement (% of the mean), MDC95%: Minimal Detectable Change at 95% confidence (% of the mean), CV%: Coefficient of Variation expressed as a percentage.

**Table A2:** Outlier screening and distribution analysis of standardized postural stability metrics.

		Median	Min	Max	Skewness	Kurtosis
Acc-AP	Floor	0.087	−1.738	2.137	0.351	2.549
	Foam Pad	−0.110	−1.684	2.985	0.948	4.342
	Rotating Disc	0.328	−1.698	1.676	−0.151	1.760
	Air Disc	−0.258	−1.821	2.068	0.312	2.368
	BOSU	−0.041	−1.847	1.589	0.160	1.964
Acc-ML	Floor	−0.250	−1.278	2.586	1.117	3.478
	Foam Pad	−0.314	−1.156	3.304	1.697	6.005
	Rotating Disc	−0.246	−1.357	2.694	1.027	3.492
	Air Disc	0.002	−1.782	2.148	0.294	2.499
	BOSU	0.120	−1.982	1.493	−0.284	2.209
CoP-AP	Floor	0.117	−1.901	2.337	0.247	2.574
	Foam Pad	−0.017	−1.595	2.061	0.333	2.471
	Rotating Disc	−0.263	−1.846	2.721	0.849	3.758
	Air Disc	0.026	−1.960	2.013	−0.223	2.620
	BOSU	−0.054	−1.693	1.810	0.232	2.192
CoP-ML	Floor	−0.021	−2.075	1.949	0.138	2.506
	Foam Pad	0.030	−2.079	1.914	−0.183	2.447
	Rotating Disc	−0.113	−1.606	2.106	0.413	2.174
	Air Disc	0.066	−2.045	2.098	0.025	2.673
	BOSU	0.022	−1.961	1.721	−0.136	2.134

Note: All variables were standardized to Z-scores (Mean = 0.00, SD = 1.00). Max values exceeding |3.29| indicate potential outliers. Skewness and Kurtosis values within |2.0| and |7.0|, respectively, support the use of parametric statistics.

**Table A3:** Trend analysis (Polynomial Contrasts) for the postural challenge graduation.

Metric	Contrast Type	F (1, 24)	*p*-Value	Partial η^2^
Acc_AP	Linear	152.170	<0.001	0.864
	Quadratic	43.533	<0.001	0.645
Acc_ML	Linear	162.938	<0.001	0.872
	Quadratic	12.437	0.002	0.341
CoP_AP	Linear	127.038	<0.001	0.841
	Quadratic	6.094	0.21	0.202
CoP_ML	Linear	78.086	<0.001	0.765
	Quadratic	8.628	0.007	0.264

**Table A4:** Planned Repeated Contrasts for Sequential Graduation Transitions across all stability metrics. Increasing order of postural challenge level: Floor, Foam Pad, Rotating Disc, Air Disc, BOSU.

Metric	Transition	*p* Value and Partial η^2^ of the Surface Pairwise Comparison			Mean Transitional Increase	
		F (1, 24)	*p*-Value *	Partial η^2^	Absolute Unit	Times of 1st Surface (*p*-Value) **
Acc_AP	Floor vs. Foam Pad	20.15	0.002	0.46	+5.603 *	+0.18
	Foam Pad vs. Rotating Disc	30.13	<0.001	0.56	+7.313 *	+0.20 (*p* = 0.692)
	Rotating Disc vs. Air Disc	59.89	<0.001	0.71	+13.968 *	+0.18 (*p* = 0.698)
	Air Disc vs. BOSU	60.69	<0.001	0.72	+14.273 *	+0.23 (*p* = 0.462)
Acc_ML	Floor vs. Foam Pad	31.63	<0.001	0.57	+3.712 *	+0.17
	Foam Pad vs. Rotating Disc	54.67	<0.001	0.69	+13.824 *	+0.37 (*p* = 0.070)
	Rotating Disc vs. Air Disc	40.87	<0.001	0.63	+11.463 *	+0.30 (*p* = 0.627)
	Air Disc vs. BOSU	50.83	<0.001	0.68	+13.543 *	+0.19 (*p* = 0.692)
CoP_AP	Floor vs. Foam Pad	15.24	<0.001	0.39	+0.084 *	+0.55
	Foam Pad vs. Rotating Disc	26.83	<0.001	0.53	+0.121 *	+0.65 (*p* = 0.677)
	Rotating Disc vs. Air Disc	11.42	<0.001	0.32	+0.113 *	+0.57 (*p* = 0.747)
	Air Disc vs. BOSU	31.69	<0.001	0.57	+0.198 *	+0.36 (*p* = 0.066)
CoP_ML	Floor vs. Foam Pad	9.79	<0.001	0.29	+0.084	+0.78
	Foam Pad vs. Rotating Disc	31.65	<0.001	0.57	+0.242 *	+0.86 (*p* = 0.924)
	Rotating Disc vs. Air Disc	27.77	<0.001	0.54	+0.267 *	+0.32 (*p* = 0.002)
	Air Disc vs. BOSU	17.55	0.003	0.42	+0.224 *	+0.27 (*p* = 0.421)

* significant difference between surfaces at *a* ≤ 0.05, ** comparison of successive transitional increases expressed as times of 1st surface, and *p*-values in parentheses denote the significance between the specific increase rising and the previous one. Except for the significant difference between the transitional rising from level 2 to level 3 (from Foam Pad to Rotating Disc) and the rising from level 3 to level 4 (from Rotating Dics to Air Disc), all progressive pairwise transitional risings were similar (*p* < 0.05), which supports the use of regression with the surface progression as the independent variable.
